# Gut microbiota composition correlates with insomnia severity: insights from high-throughput sequencing analysis

**DOI:** 10.3389/fmicb.2025.1733772

**Published:** 2026-01-14

**Authors:** Zhe Xu, Jinxia Zhang, Haifang Zhou, Lei Zhang, Xiuhong Zhang

**Affiliations:** 1 Sleep Medicine Center, Hangzhou Hospital of Traditional Chinese Medicine Affiliated to Zhejiang Chinese Medical University, Hangzhou, Zhejiang, China; 2 Department of Nursing, Hangzhou Hospital of Traditional Chinese Medicine Affiliated to Zhejiang Chinese Medical University, Hangzhou, Zhejiang, China

**Keywords:** 16S rDNA, gut microbiota, insomnia, insomnia severity, microbiota-gut-brain axis

## Abstract

**Objective:**

This study investigates differential alterations in gut microbiota characteristics across varying degrees of insomnia severity, aiming to provide empirical evidence elucidating the relationship between sleep disorders and the gut microbiome.

**Methods:**

A total of 120 insomnia patients, treated at the Clinical Psychology Department of Hangzhou Hospital of Traditional Chinese Medicine between October 2023 and May 2024, were enrolled and categorized into mild (Group B, *n* = 16), moderate (Group C, *n* = 42), and severe (Group D, *n* = 42) cohorts based on Pittsburgh Sleep Quality Index (PSQI) scores. A control group of 20 healthy volunteers (Group A) was recruited for comparison. Fecal samples were collected from all participants for gut microbiota DNA extraction. High-throughput sequencing of the 16S rDNA was performed on the Illumina NovaSeq platform to analyze the alpha diversity, beta diversity, and species composition of the gut microbiota across the groups.

**Results:**

Alpha diversity analysis revealed significantly elevated Chao1 and ACE indices in the insomnia cohorts compared to controls (*p* < 0.01). Notably, the Shannon index was significantly elevated only in the mild group (Group B, *p* < 0.05). Beta diversity analysis indicated significant differences in the microbial community structure among groups, with a gradient shift corresponding to increasing PSQI scores. Compositional analysis revealed a progressive decline in the Firmicutes/Bacteroidetes (F/B) ratio that paralleled increasing insomnia severity. The abundance of *Clostridia* demonstrated a graded decline with increasing insomnia severity, with a significant reduction of approximately 15% observed even in the mild sleep disorder group. LEfSe analysis identified distinct differential microbiota for each group: the healthy group was characterized by Firmicutes and *Akkermansia*, while the sleep disorder groups were, respectively, enriched in *Fusobacteriota*, *Cyanobacteria*, and Desulfobacterota.

**Conclusion:**

Gut microbiota structure is intrinsically linked to insomnia severity, characterized by a diminished F/B ratio, reduced *Clostridia* abundance, and enrichment of pro-inflammatory taxa. The reduction in SCFA-producing *Clostridia* may impair neuro-immune regulation via the gut-brain axis, potentially exacerbating sleep dysregulation.

## Highlights

- Gut microbiota structure exhibits a graded shift corresponding to insomnia severity.
- Microbial richness peaks in mild insomnia, suggesting a non-linear relationship.
- The Firmicutes/Bacteroidetes ratio and SCFA-producing *Clostridia* decrease as insomnia worsens.
- Pro-inflammatory bacteria are progressively enriched with increasing insomnia severity.

## 1 Introduction

Emerging evidence highlights the pivotal role of the gut microbiota in regulating brain function, emotion, and behavior via the bidirectional “Microbiota-Gut-Brain (MGB) Axis” (Cryan et al., 2019). Studies involving germ-free animals have been pivotal in establishing the link between the gut microbiota and brain development and function (Bercik et al., 2011; Neufeld et al., 2011; Poroyko et al., 2016; Bowers et al., 2020; Lu et al., 2018). Substantial clinical evidence suggests that human gut microbiota composition correlates with sleep quality. Through neural, endocrine, immune, and metabolic pathways, the microbiome forms a complex regulatory network with the central nervous system (CNS), acting as a critical modulator of sleep physiology (Wang et al., 2022; Wang et al., 2025). Prospective animal experiments have found that after sleep restriction and fragmentation interventions in mice, both the richness and diversity of the gut microbiota significantly decreased (Poroyko et al., 2016; Bowers et al., 2020). Clinical cohort studies have also discovered significant alterations in the Firmicutes/Bacteroidetes (F/B) ratio in the gut of insomnia patients, suggesting a close connection between changes in microbial structure and sleep disorders (Grosicki et al., 2020; Wang et al., 2022; Li et al., 2024b). Mechanistically, gut microbial metabolites, particularly short-chain fatty acids (SCFAs), have been shown to modulate the hypothalamic–pituitary–adrenal (HPA) axis via vagus nerve stimulation. This interaction influences neuronal activity in sleep-regulating regions, such as the suprachiasmatic nucleus, thereby affecting the sleep–wake cycle (Silva et al., 2020; Wang et al., 2022). The gut microbiota also directly synthesizes or regulates neurotransmitters like serotonin (5-HT), gamma-aminobutyric acid (GABA), and melatonin. Approximately 90% of 5-HT is synthesized under the mediation of the gut microbiota, and abnormalities in the metabolism of its precursor, tryptophan, are closely related to insomnia and mood disorders (Wang et al., 2022).

Despite growing interest, research into the insomnia-microbiota nexus remains in its infancy. Existing studies predominantly rely on binary comparisons between “insomnia” and “healthy” states, often overlooking the nuanced microbial alterations corresponding to varying degrees of insomnia severity. Therefore, this study aims to explore the differential shifts across a spectrum of insomnia severity. By doing so, we seek to provide microbiological insights into the taxonomic signatures and ecological shifts underlying the gut-brain axis in sleep disorders.

## 2 Methods

### 2.1 Study population and design

This study enrolled patients with insomnia from the outpatient and inpatient departments of the Clinical Psychology Department at Hangzhou Hospital of Traditional Chinese Medicine between October 2023 and May 2024. These patients constituted the observation groups. Based on their Pittsburgh Sleep Quality Index (PSQI) scores, they were divided into a mild sleep disorder group (Group B), a moderate sleep disorder group (Group C), and a severe sleep disorder group (Group D). Concurrently, 20 healthy volunteers were recruited from the health examination center to serve as the control group (Group A). To minimize environmental and dietary confounders, these participants were matched with the observation groups in terms of age, gender, and geographical residence (Hangzhou area). All participants maintained a standard local diet without specific restrictions (e.g., vegetarianism or ketogenic diet), and those with extreme dietary habits were excluded. All procedures were approved by the Ethics Committee of Hangzhou TCM Hospital Affiliated with Zhejiang Chinese Medical University (Approval No.2023KLL020). Ethical approval in accordance with the Declaration of Helsinki. Participants provided written consent, and data were anonymized.

### 2.2 Inclusion and exclusion criteria

Patients were enrolled if they met the following criteria: (1) Age: Between 18 and 60 years; (2) Diagnosis: Fulfilled the diagnostic criteria for non-organic insomnia (Code F51.0) as defined by the International Classification of Diseases, 10th Revision (ICD-10); (3) Compliance: Demonstrated the capacity for strict adherence to study protocols, including the completion of questionnaires and standardized fecal sampling. Healthy volunteers were included if they were aged 18–60 years, in good physical health with unremarkable physical examination results, and reported no history of insomnia (PSQI score < 5). Additionally, participants were required to have no symptoms of anxiety or depression (SDS and SAS standard scores ≤ 60) and provided written informed consent. All procedures adhered to the Declaration of Helsinki, and written informed consent was obtained from all subjects prior to enrollment.

Participants were excluded if they presented with any of the following conditions: (1) Severe Systemic Pathology: Severe dysfunction of major organ systems, including cardiovascular, cerebrovascular, hepatic, renal, or hematopoietic disorders. (2) Psychiatric Comorbidities: Comorbid severe psychiatric disorders, such as schizophrenia or dissociative (conversion) disorders. (3) Surgical History: History of major gastrointestinal surgery (e.g., cholecystectomy, appendectomy) or significant intestinal resection within the preceding 5 years. (4) Gastrointestinal Disorders: Diagnosed inflammatory bowel disease (IBD; including ulcerative colitis, Crohn’s disease, or indeterminate colitis), irritable bowel syndrome (IBS), or persistent infectious gastroenteritis. (5) Chronic GI Symptoms/Infection: Unexplained chronic diarrhea or untreated *Helicobacter pylori* infection. (6) GI Lesions or Malignancy: History of gastrointestinal ulcers, hemorrhage, polyps, masses, or malignancy. (7) Medication Use: Administration of antibiotics, bismuth subsalicylate, or other microbiome-altering agents within 3 months prior to sampling.

### 2.3 Data collection and sleep assessment

Baseline information, including gender, age, education level, present illness, history, and family history, was provided by the subjects or their families. Sleep quality was assessed using the Pittsburgh Sleep Quality Index (PSQI), which comprises seven factors: subjective sleep quality, sleep latency, sleep duration, sleep efficiency, sleep disturbances, use of sleeping medication, and daytime dysfunction. The total PSQI score ranges from 0 to 21, with higher scores indicating poorer sleep quality. Severity was graded according to the “Chinese Guidelines for the Diagnosis and Treatment of Adult Insomnia” and PSQI scores: mild sleep disorder (Group B, PSQI 6–10), moderate sleep disorder (Group C, PSQI 11–15), and severe sleep disorder (Group D, PSQI 16–21). Healthy volunteers with a PSQI score ≤ 5 were included as the control group (Group A).

### 2.4 Fecal sample collection and processing

A standardized protocol was used for all sample collections: (1) Participants were instructed to wash their hands thoroughly with soap or hand sanitizer. (2) Urination was completed before defecation to avoid contamination of the fecal specimen. (3) To prevent contamination, a sterile collection sheet was utilized. (4) A sterile spoon was used to collect a mid-stream portion of the stool that had not been exposed to air or the toilet surface. Approximately 200 mg of fecal matter (equivalent to three soybean-sized aliquots) was collected into sterile cryotubes. (5) The tube was tightly sealed and stored in a −80 °C freezer, avoiding repeated freeze–thaw cycles. Samples were transported on dry ice. (6) Each sample was clearly labeled with the patient’s information, including sample ID, name, sample type, and collection time.

### 2.5 DNA extraction and 16S rDNA sequencing

Total DNA from the fecal microbiome was extracted using the OMEGA-soil DNA kit (M5636-02). DNA purity and concentration were measured using a NanoDrop NC2000 spectrophotometer, and DNA integrity was assessed by agarose gel electrophoresis. The V3-V4 hypervariable region of the bacterial 16S rDNA gene was amplified by PCR using primers 341F (5’-CCTACGGGNGGCWGCAG-3′) and 805R (5’-GACTACHVGGGTATCTAATCC-3′). The PCR program consisted of an initial denaturation at 98 °C for 30s, followed by 35 cycles of denaturation at 98 °C for 10s, annealing at 53 °C for 30s, and extension at 72 °C for 45 s, with a final extension at 72 °C for 10 min. The PCR products were confirmed by 2% agarose gel electrophoresis, purified with AMPure XT beads, and quantified using Qubit. Amplicons were pooled in equimolar amounts and sequenced on an Illumina NovaSeq platform using a paired-end 250 bp (PE250) high-throughput sequencing mode.

### 2.6 Bioinformatic and statistical analysis

Raw sequencing data were filtered and processed using the QIIME2 DADA2 pipeline to generate Amplicon Sequence Variants (ASVs). Species annotation was performed for each characteristic ASV sequence. Alpha diversity, which reflects the species richness and evenness within a given ecosystem, was evaluated using a comprehensive set of indices. Specifically, community richness was estimated using the Chao1 and Observed Species indices, while microbial diversity was assessed via the Shannon and Simpson indices. Additionally, Faith’s Phylogenetic Diversity (PD) index was employed to capture phylogenetic breadth, and Pielou’s evenness index was used to measure species distribution uniformity. Sequencing depth sufficiency was verified using Good’s coverage index and visualized through rarefaction curves. Beta diversity was evaluated based on distance matrices calculated at the ASV level. Principal Coordinate Analysis (PCoA) using Bray–Curtis dissimilarity was employed to visualize community structural differences, with statistical significance determined by Permutational Multivariate Analysis of Variance (PERMANOVA). To further identify specific bacterial taxa driving these differences, Linear Discriminant Analysis Effect Size (LEfSe) was performed, and the effect size of discriminative features was estimated using LDA scores.

Statistical analysis was performed using IBM SPSS 25.0. Baseline data are presented as mean ± standard deviation (x̄ ± s). One-way ANOVA was used for overall comparisons among multiple groups; Welch’s ANOVA was used if variances were unequal. The Kruskal-Wallis H test was used for non-normally distributed variables. If the overall test was significant, appropriate post-hoc tests were conducted for pairwise comparisons. The chi-square test, or Fisher’s exact test when necessary, was used for categorical variables. A *p*-value < 0.05 was considered statistically significant.

## 3 Results

### 3.1 General characteristics

A total of 120 patients with insomnia and 20 healthy volunteers (Group A) were included. The insomnia patients were divided into Group B (mild, *n* = 16), Group C (moderate, *n* = 42), and Group D (severe, *n* = 42). The mean ages were 47.58 ± 12.32, 46.04 ± 11.67, 49.58 ± 9.41, and 46.58 ± 10.61 years for Groups A, B, C, and D, respectively. No statistically significant differences were observed among the four cohorts regarding demographic characteristics (gender, age, education) or physiological indices (BMI, blood pressure), ensuring baseline comparability (*p* > 0.05). The mean PSQI total scores were 2.72 ± 1.02, 9.15 ± 1.75, 14.35 ± 2.10, and 19.76 ± 1.12 for Groups A, B, C, and D, respectively, showing a highly significant difference (*p <* 0.001).

**Figure 1:**
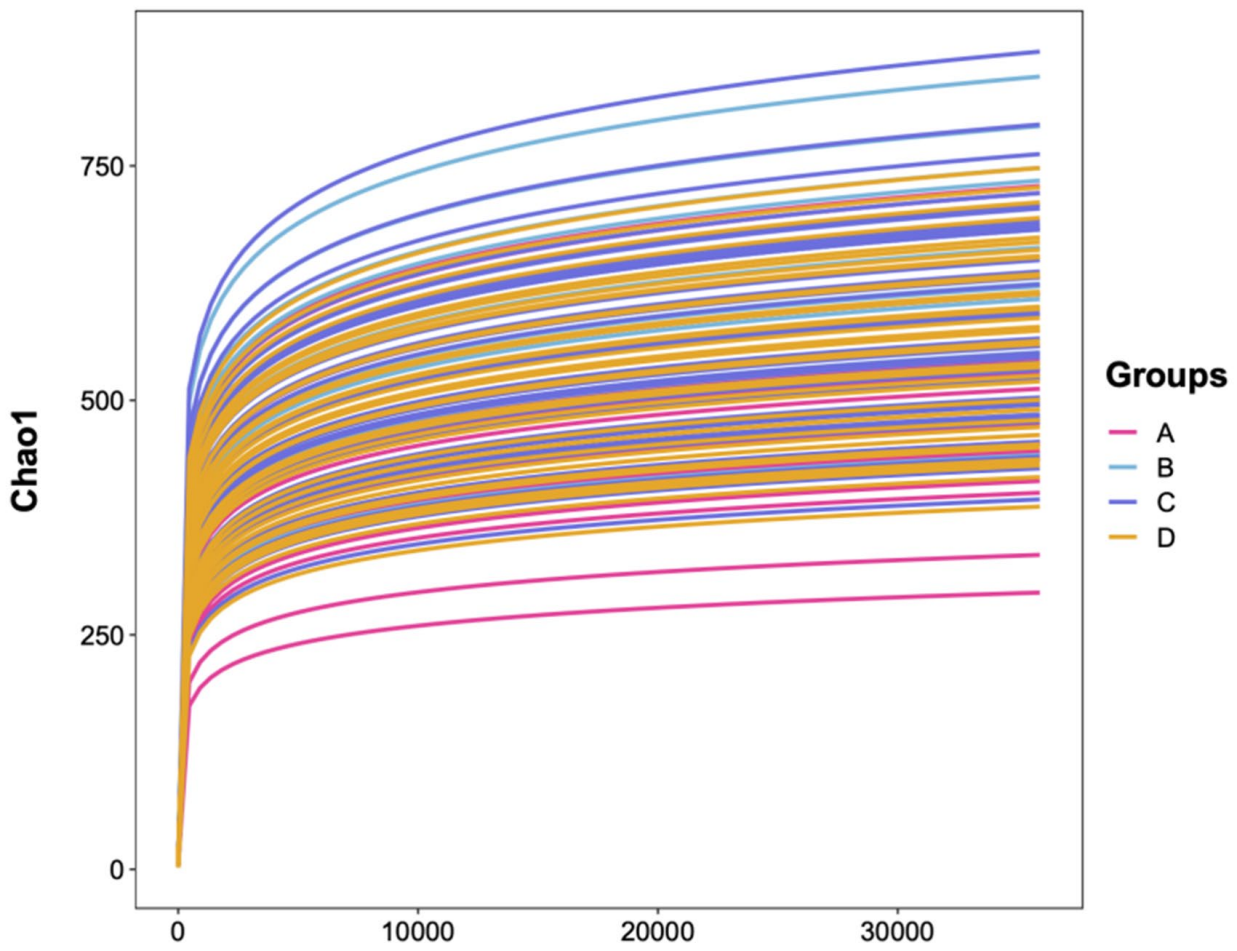
Species accumulation curve.

The species accumulation curves for all groups gradually plateaued with increasing sequencing depth, indicating that the sequencing depth and sample size were sufficient to cover the majority of species. The sequencing data were reliable for subsequent analysis. Good’s coverage analysis showed that coverage was close to 1 (>0.99) for all groups, confirming adequate sequencing depth (Figure 1).

### 3.2 Alpha diversity analysis

**Figure 2:**
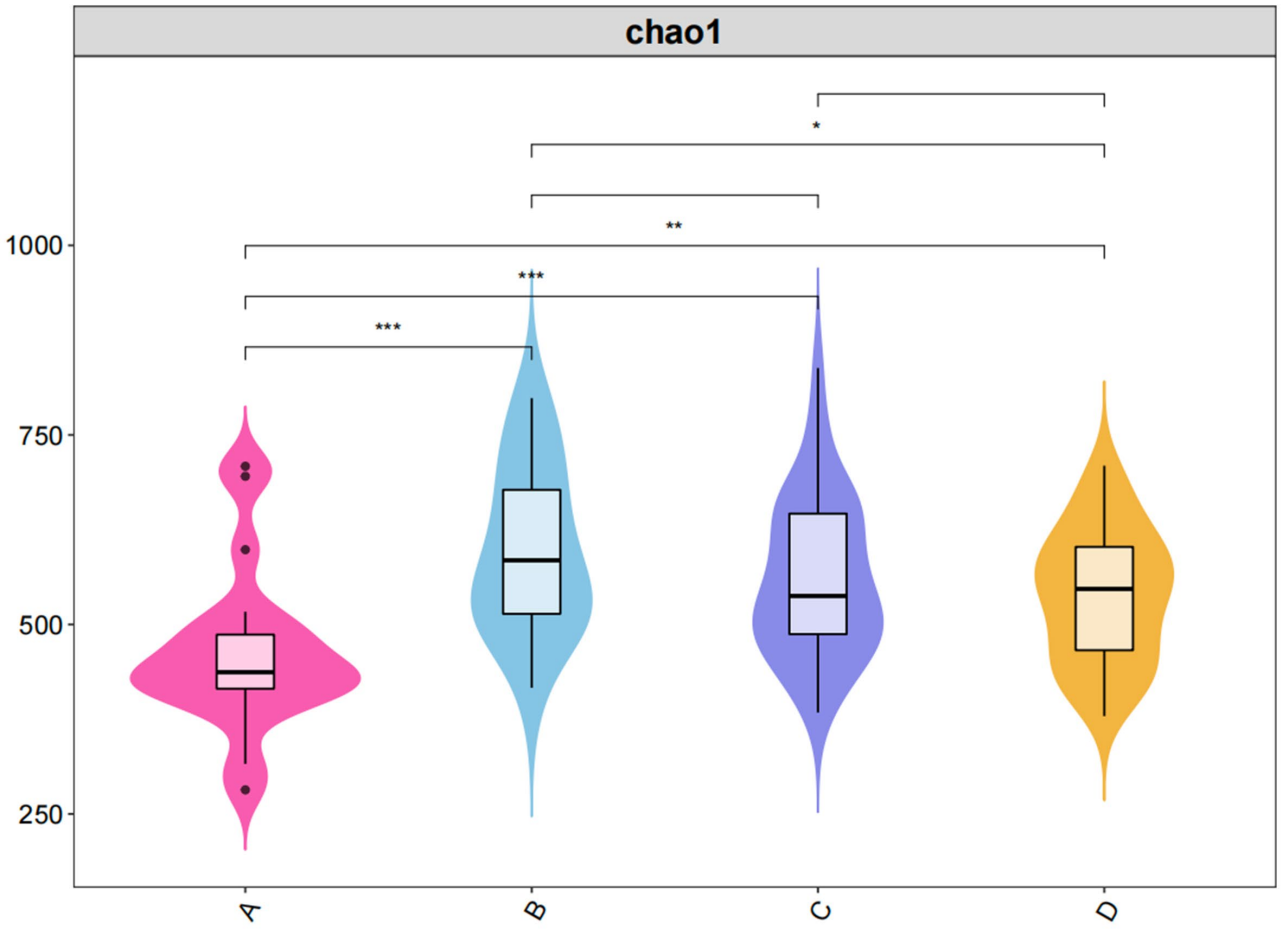
Violin plot of Chao1 index.

Alpha diversity analysis revealed marked inter-group heterogeneity in community richness, as evidenced by Chao1 and ACE indices (*p* < 0.001). Group B exhibited the highest values for most alpha diversity metrics, while Group A generally had the lowest. Specifically, the Shannon diversity index was highest in Group B (6.38 ± 0.56) and lowest in Group A (5.40 ± 0.58). The Simpson index was also highest in Group B (0.95 ± 0.02) and lowest in Group A (0.87 ± 0.19). The Chao1 richness index was highest in Group B (610.82 ± 91.61) and lowest in Group A (407.12 ± 90.33). Pielou’s evenness index was highest in Group B (0.77 ± 0.08) and lowest in Group C (0.64 ± 0.08) (Table 1; Figure 2).

**Table 1:** Alpha diversity indices of the gut microbiota (Mean ± SD).

Indices	Group A	Group B	Group C	Group D
Shannon	5.40 ± 0.58	6.38 ± 0.56^**^	6.08 ± 1.04	6.00 ± 1.11
Simpson	0.87 ± 0.19	0.95 ± 0.02	0.92 ± 0.07	0.88 ± 0.09
Chao1	407.12 ± 90.33	610.82 ± 91.61^***^	597.10 ± 75.43^***^	531.11 ± 93.30^**^
Goods_Coverage	1.00 ± 0.00	1.00 ± 0.00	1.00 ± 0.00	1.00 ± 0.00
Pielou_E	0.65 ± 0.10	0.77 ± 0.08	0.64 ± 0.08	0.67 ± 0.10
ACE	447.62 ± 83.41	627.10 ± 109.54^***^	565.70 ± 83.82^***^	551.00 ± 93.42^**^

*p < 0.05, **p < 0.01, ***p < 0.001 vs. Group A.

### 3.3 Beta diversity analysis

PCoA was conducted using four different distance algorithms to assess inter-group structural differences in the microbiota.

#### 3.3.1 Overall distribution

**Figure 3:**
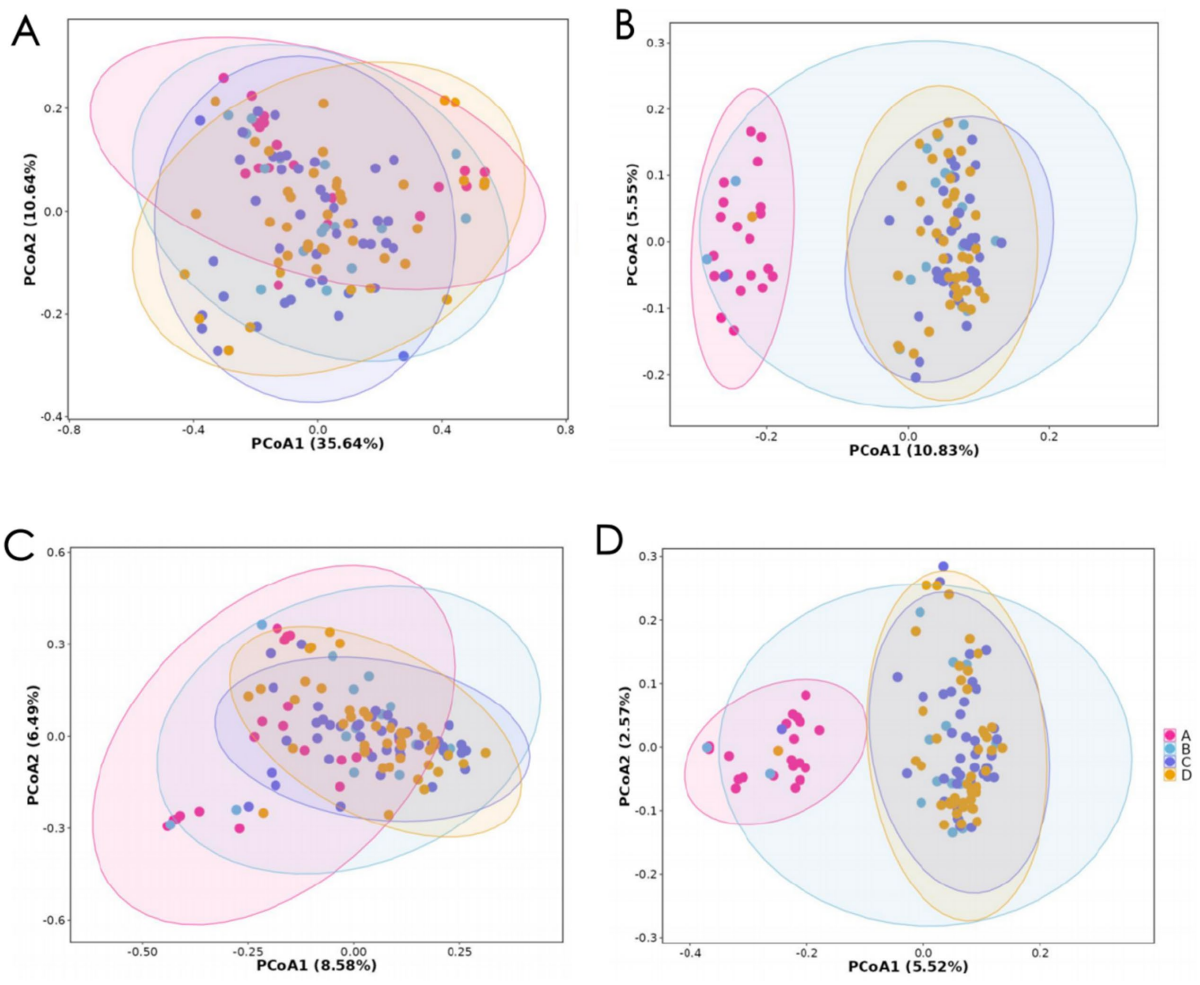
Principal coordinate analysis (PCoA) plot. **(A)** Weighted unifrac NMDS; **(B)** unweighted unifrac NMDS; **(C)** Bray-Curtis NMDS; **(D)** Jaccard NMDS.

Principal Coordinate Analysis (PCoA) demonstrated distinct clustering patterns. The confidence ellipses for the control group (Group A) were spatially distinct from the insomnia cohorts. Notably, a stepwise gradient shift in microbial community structure was observed corresponding to increasing PSQI scores (B → C → D). In the weighted_unifrac plot (Figure 3A), Group C (purple) and Group D (yellow) were distinctly separated along the PCoA1 axis (explaining 35.64% of the variance), suggesting that the most significant structural changes in microbiota occur in moderate to severe sleep disorders.

#### 3.3.2 Sensitivity of different algorithms

The weighted_unifrac metric (Figure 3A) had the highest cumulative explained variance (PCoA1 35.64% + PCoA2 10.64%), showing the clearest separation between groups, especially with Group A having the least overlap. This indicates that this method effectively captures phylogenetic information related to sleep disorders (*R =* 0.0252, *p <* 0.05). The unweighted unifrac metric (Figure 3B) had the lowest explained variance (PCoA1 10.83% + PCoA2 5.55%), with considerable overlap among groups, suggesting that considering only the presence/absence of species is less sensitive to sleep-related microbial changes (*R =* 0.34, *p <* 0.001). Bray-Curtis (Figure 3C) and Jaccard (Figure 3D) showed moderate discriminatory power, with some overlap but still discernible distribution trends (*R =* 0.11, *p <* 0.001; *R =* 0.34, *p <* 0.001, respectively).

### 3.4 Gut microbiota composition analysis

#### 3.4.1 Phylum level

**Figure 4:**
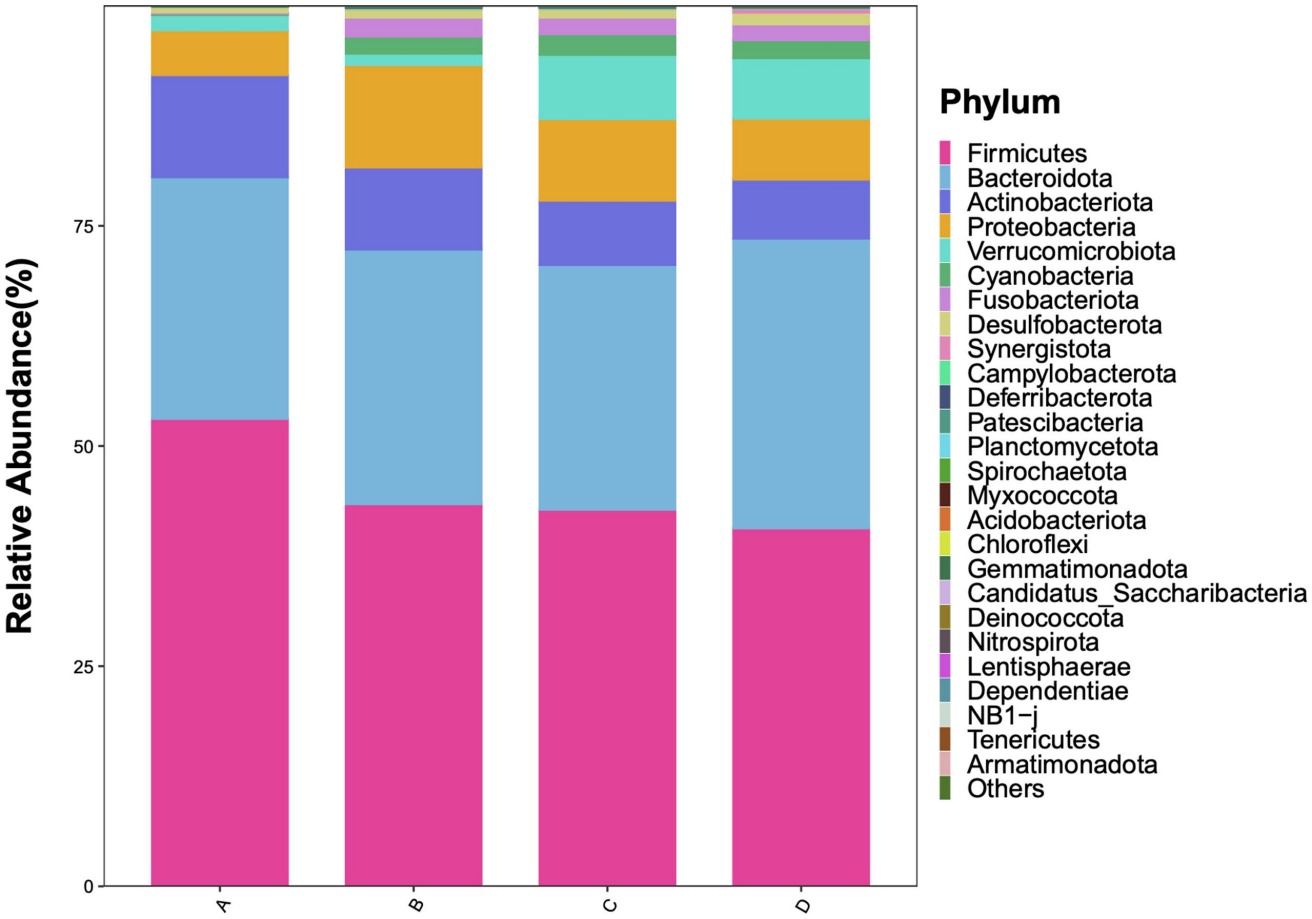
Relative abundance of gut microbiota at the phylum level.

The abundance of Firmicutes was positively correlated with sleep quality. In the healthy control group, Firmicutes abundance was over 75%, whereas it dropped to around 40% in the severe sleep disorder group. The Firmicutes/Bacteroidetes (F/B) ratio was highest in the control group (Group A) and gradually decreased in the observation groups as sleep disorders worsened (B → D). Actinobacteriota was significantly enriched in the healthy population, while Verrucomicrobiota was detected at higher abundances in patients with moderate to severe insomnia (Figure 4).

#### 3.4.2 Class level

**Figure 5:**
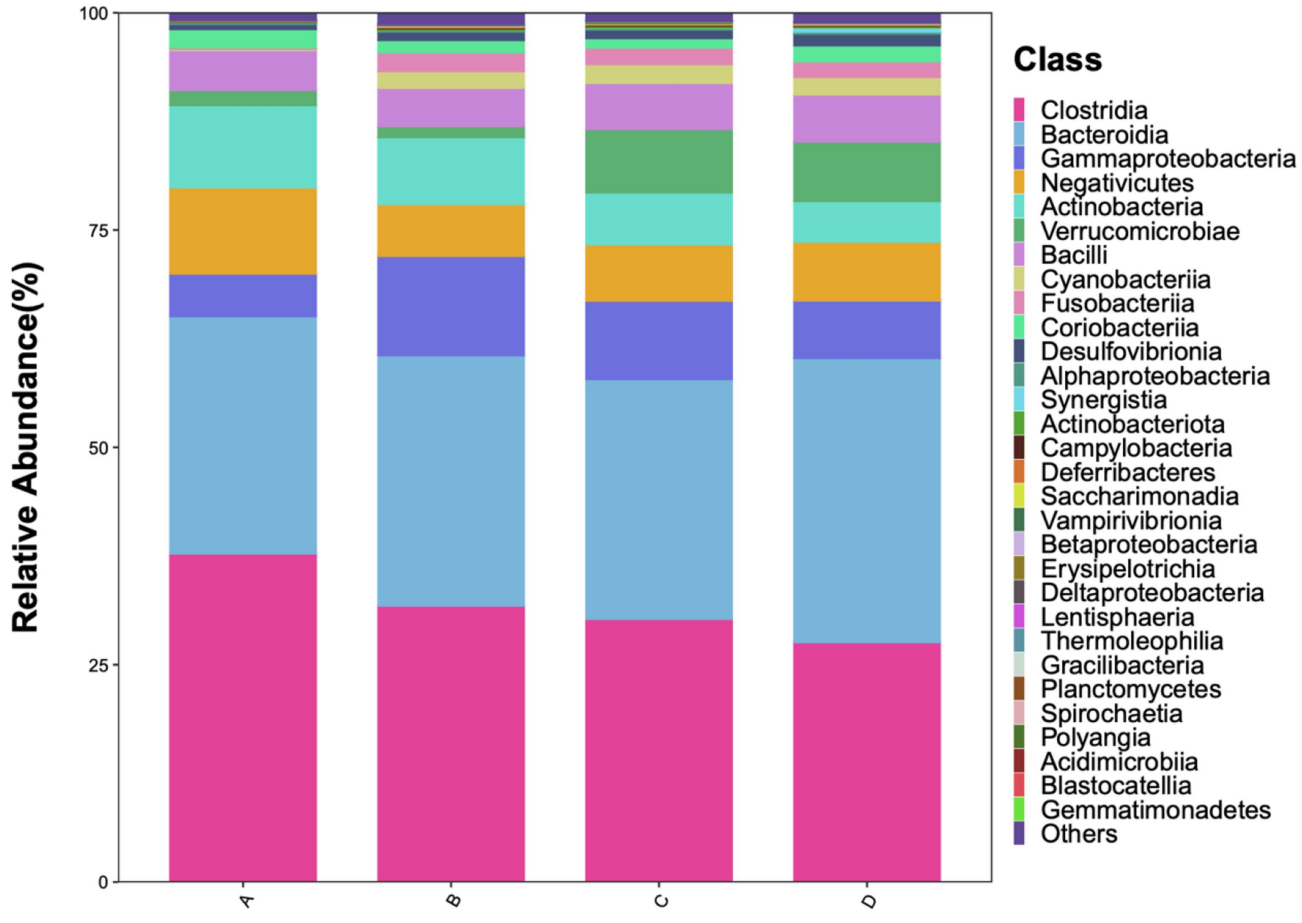
Relative abundance of gut microbiota at the class level.

*Clostridia* and *Bacteroidia* were the dominant classes in all four groups, especially in Group A. The abundance of *Clostridia* showed a graded decrease with increasing severity of sleep disorder. Specifically, a precipitous decline of approximately 15% was observed even in the mild insomnia cohort compared to controls. The abundance of *Gammaproteobacteria* was significantly higher in the sleep disorder groups. The abundance of *Actinobacteria* also showed a graded decrease with worsening sleep disorders. The abundance of *Verrucomicrobiae* was significantly lower in Groups A and B compared to Groups C and D (*p <* 0.01). Bacilli were present in all samples with no significant differences. The abundances of *Cyanobacteria* and *Fusobacteriia*, though low in all groups, were significantly increased in the sleep disorder groups (*p <* 0.01) (Figure 5).

### 3.5 Analysis of key differential microbiota

**Figure 6:**
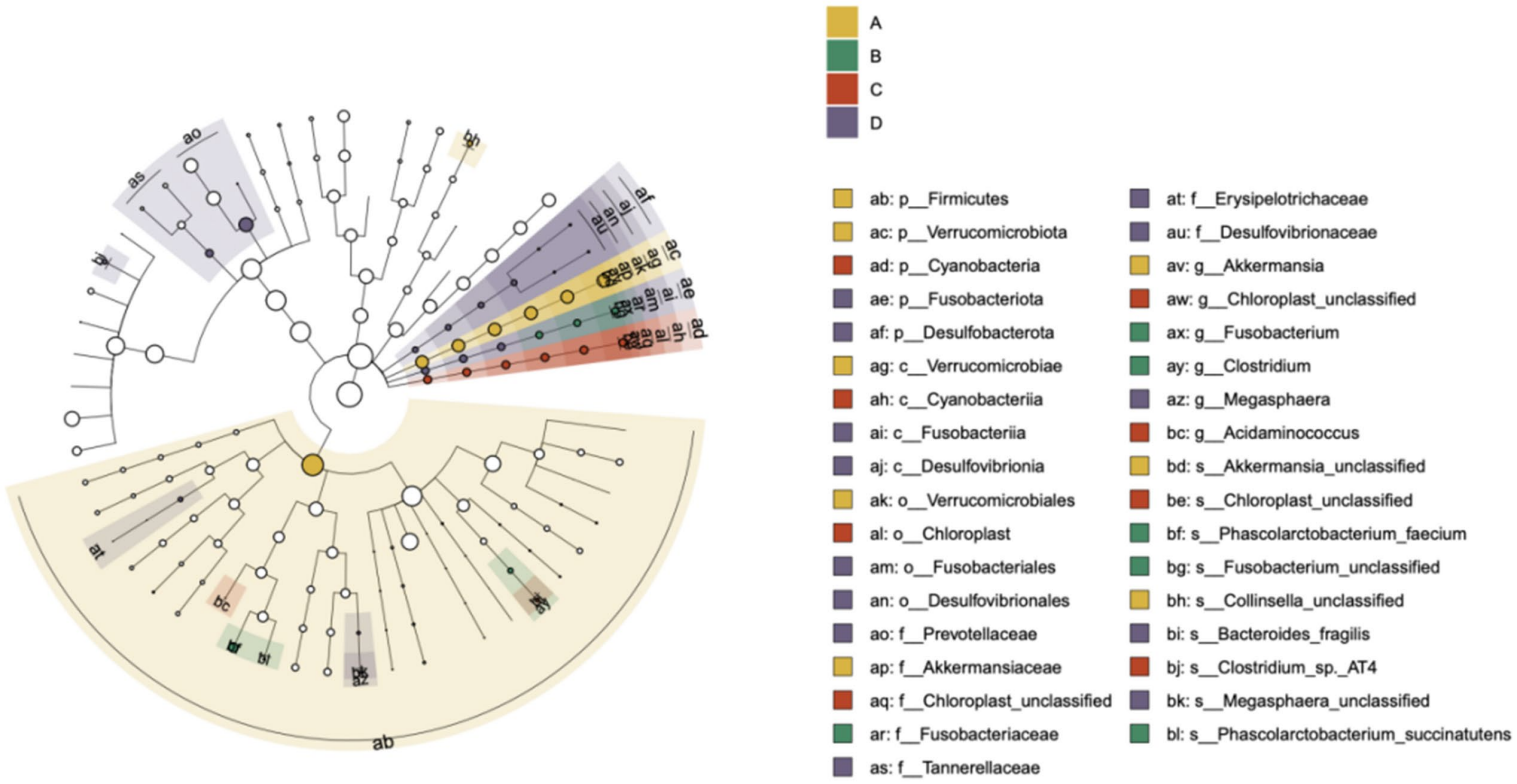
Cladogram from LEfSe analysis showing the taxonomic biomarkers differentiating the healthy controls and the insomnia patients.

LEfSe analysis was employed to identify key differential bacterial taxa. The core microbiota characterizing Group A (yellow) were Firmicutes, *Akkermansia*, and *Verrucomicrobiota*. Group B (green) was characterized by *Fusobacteriota* and its subordinate taxa. Group C (red) was characterized by *Cyanobacteria* and related taxa. Group D (purple) was characterized by Desulfobacterota, particularly the class Desulfovibrionia (Figure 6).

## 4 Discussion

Our findings suggest a potential non-linear relationship between the severity of sleep disorders and gut microbiota diversity. Contrary to the assumption that higher diversity always equates to better health, the healthy controls (Group A) exhibited lower Alpha diversity (Chao1/ACE) compared to the mild insomnia group (Group B). We posit that the microbial richness in healthy controls reflects a stable ‘homeostatic baseline’ maintained by competitive exclusion among core commensals. The observed peak in diversity within the mild insomnia group aligns with the ‘Intermediate Disturbance Hypothesis’ (IDH) (Connell and Connell, 1978). This ecological theory posits that species diversity is maximized at intermediate levels of disturbance. In the context of sleep pathology, the onset of mild insomnia may act as a moderate physiological stressor (Matenchuk et al., 2020). In the context of sleep pathology, mild insomnia may function as a moderate physiological stressor. This perturbation likely disrupts the competitive dominance of core taxa, permitting a transient proliferation of opportunistic species or compensatory diversification to preserve functional redundancy (Coyte et al., 2015). However, under chronic and severe stress (Groups C and D), the ecosystem’s resilience threshold is likely exceeded, precipitating the observed collapse in diversity (Sommer et al., 2017). This non-linear dynamic is corroborated by recent studies indicating that microbial richness can spike during the early stages of sleep disruption (Holzhausen et al., 2024). Moreover, the observation that effective interventions return this elevated diversity to baseline levels further supports the hypothesis that the initial increase is a compensatory mechanism (Li et al., 2024a).

The alpha diversity indices (Chao1/ACE) were significantly higher in all sleep disorder groups and exhibited a stepwise decrease, peaking in the mild sleep disorder group and then declining as the disorder’s severity increased. PCoA-based beta diversity analysis also revealed a graded shift in community structure corresponding to the worsening of sleep disorders. This is consistent with a study by Li et al., which found in a randomized controlled trial that a high-dose probiotic group had the most significant decrease in PSQI scores and a corresponding decrease in alpha diversity (Chao1 index). Other research has also noted that the Chao1 index is significantly higher in individuals with mild sleep disturbances (PSQI 5–7) compared to healthy controls, suggesting the microbiota might increase diversity to buffer stress (Li et al., 2024a). Conversely, in severe sleep disorders (sleep efficiency <85% and WASO >60 min), the Chao1 index decreased by 22% (*p <* 0.01), as inflammation and metabolic stress led to a substantial loss of diversity (Wang et al., 2022; Holzhausen et al., 2024). This aligns with the stepwise decline in Chao1/ACE observed in our study, supporting the possibility of a compensatory regulatory mechanism in the gut microbiota. The lack of significant difference in the Shannon index between the sleep disorder groups and the control group suggests that insomnia is primarily associated with changes in microbial richness rather than evenness. For instance, a large observational study showed that variability in nightly sleep duration, increased wake time, and reduced sleep efficiency were significantly associated with lower Chao1 richness but only weakly linked to the Shannon index (Holzhausen et al., 2024). Our diversity analysis and evidence from multiple studies reveal a potential non-linear relationship, but further research into specific molecular pathways (e.g., the SCFA-GPCR axis, immune modulation, or neurotransmitter regulation) is needed to fully substantiate these findings (Smith et al., 2019; Li et al., 2022; Holzhausen et al., 2024).

The gut microbiota structure of patients with sleep disorders exhibits distinct features compared to healthy individuals. Our analysis at the phylum and class levels, along with LEfSe analysis, revealed that the gut microbiota of healthy controls is dominated by Firmicutes (class *Clostridia*) and Bacteroidetes (class *Bacteroidia*). As the severity of sleep disorders increased, the abundance of these dominant taxa and the F/B ratio progressively decreased (Wang et al., 2022). This is in line with findings from Xu et al. (2017) and Dongyang et al. (2024), who reported a significantly lower F/B ratio and *Clostridia* abundance in individuals with chronic insomnia, negatively correlating with disorder severity. It is well-established that *Akkermansia*, a representative genus of the phylum *Verrucomicrobiota*, plays a crucial role in maintaining gut barrier integrity. Our findings, however, present a paradoxical trend: *Verrucomicrobiota* was detected at a notable abundance in patients with moderate-to-severe insomnia, and its levels increased as the severity of the disorder decreased. This echoes a similar paradoxical observation by Yang et al., who also reported elevated levels of *Akkermansia* in the fecal samples of stroke patients (Ma et al., 2025). We postulate that this may represent a compensatory mechanism, wherein *Verrucomicrobiota* proliferates to enhance gut barrier function in response to sleep disturbances. Nevertheless, further research is required to fully elucidate this apparent contradiction. In summary, the gut microbiota structure in patients with sleep disorders is characterized by: (a) altered proportions of core dominant bacteria; (b) relative enrichment of low-abundance pro-inflammatory phyla; and (c) proliferation of opportunistic pathogens, leading to impaired ecological network stability. This study further validates the hypothesis that sleep–wake cycle dysregulation may be linked to impaired gut barrier function and an inflammatory state.

The gut microbiota may influence host sleep behavior by mediating neuro-immune function, neurotrophic factor production, and blood–brain barrier integrity via SCFAs through the gut-brain axis. Our results show that the F/B ratio and the abundance of *Clostridia* (a class within Firmicutes) decrease as insomnia severity increases, with a notable ~15% drop in *Clostridia* even in the mild sleep disorder group. As the primary producers of SCFAs (e.g., butyrate, propionate) in the gut, a reduction in *Clostridia* could directly impair SCFA-mediated neuro-immune regulation. Interestingly, another observational study reported a 2.3-fold increase in the abundance of butyrate-producing genera in patients with mild sleep disorders (Holzhausen et al., 2024). Wang et al. (2025) demonstrated that sleep deprivation reduced butyrate levels in the feces and hypothalamus of SPF mice, and oral supplementation with butyrate rescued sleep disturbances in recipient mice, indicating that the gut microbiota can influence host sleep via metabolites like butyrate. Similarly, administration of milk fermented with the high-GABA strain *Lactobacillus brevis* DL1-11 has been shown to enhance sleep quality and reduce anxiety, effects that paralleled increases in both beneficial flora and SCFA production (Yu et al., 2020). SCFAs have been shown to stimulate the production of neurotrophic factors (e.g., NGF, BDNF), promote the synthesis of neurotransmitters (e.g., glutamate, GABA), and regulate neuronal growth and excitability (van de Wouw et al., 2018; Berding et al., 2021; Frost et al., 2025). Notably, butyrate has been demonstrated in animal models to enhance blood–brain barrier integrity by increasing occludin expression, thereby protecting the brain from potential neurotoxins (Li et al., 2016; Sun et al., 2016). These findings collectively suggest a strong link between *Clostridia* abundance, SCFAs, and sleep disorders, although more multi-level research is required to strengthen the evidence chain and support deeper mechanistic investigations. It is important to interpret the causal direction of our findings with caution, particularly given the lack of strict dietary controls. While we hypothesize that microbiota alterations impact sleep via the gut-brain axis, a bidirectional relationship is plausible. Sleep deprivation is known to alter appetite-regulating hormones (e.g., ghrelin and leptin), potentially leading to increased consumption of high-calorie or high-sugar foods, which in turn reshapes the gut microbiome. Therefore, the distinct microbial signatures observed in our severe insomnia group could partially reflect sleep-loss-induced dietary changes. Future longitudinal studies incorporating detailed dietary logs are essential to disentangle these bidirectional effects.

## 5 Limitation

This study has certain limitations. As a cross-sectional study, it cannot establish a causal relationship between microbiota changes and sleep disorders. A primary limitation of this study is the imbalance in sample sizes across groups, particularly the smaller cohorts in the mild insomnia (*n* = 16) and healthy control (*n* = 20) groups compared to the moderate and severe groups. While our sample accumulation curves indicated sufficient sequencing depth, we acknowledge that the statistical power to detect subtle differences in the smaller groups may be limited. However, the observation of significant gradient trends despite these sample size constraints suggests a potentially robust association. Future studies should aim for larger, balanced cohorts to validate these gradient changes and perform *a priori* power analyses to ensure adequate sensitivity. Furthermore, this study, like many others, did not fully control for the confounding effects of diet and medications (e.g., sedatives) on the microbiota. Future research should therefore aim to increase sample sizes, incorporate standardized dietary records and metabolomic analyses, and employ longitudinal cohort studies and animal experiments to establish causality.

## Data Availability

The sequencing data presented in this study have been deposited in the National Genomics Data Center (https://ngdc.cncb.ac.cn/). The specific dataset can be accessed with the accession number CRA035674.
